# Street-Scale Analysis of Population Exposure to Light Pollution Based on Remote Sensing and Mobile Big Data—Shenzhen City as a Case

**DOI:** 10.3390/s20092728

**Published:** 2020-05-11

**Authors:** Bo Sun, Yang Zhang, Qiming Zhou, Duo Gao

**Affiliations:** 1 Shenzhen Institutes of Advanced Technology, Chinese Academy of Sciences, Shenzhen 518055, China; sunbo@siat.ac.cn (B.S.); yang.zhang2@siat.ac.cn (Y.Z.); 2 Department of Geography, Hong Kong Baptist University, KLN, Hong Kong, China; 3 TalkingData Co., Ltd., Beijing 100027, China; ted.gao@tendcloud.com

**Keywords:** light pollution, NTL remote sensing, Luojia 1-01, residential area, population exposure to light pollution

## Abstract

Most studies on light pollution are based on light intensity retrieved from nighttime light (NTL) remote sensing with less consideration of the population factors. Furthermore, the coarse spatial resolution of traditional NTL remote sensing data limits the refined applications in current smart city studies. In order to analyze the influence of light pollution on populated areas, this study proposes an index named population exposure to light pollution (PELP) and conducts a street-scale analysis to illustrate spatial variation of PELP among residential areas in cites. By taking Shenzhen city as a case, multi-source data were combined including high resolution NTL remote sensing data from the Luojia 1-01 satellite sensor, high-precision mobile big data for visualizing human activities and population distribution as well as point of interest (POI) data. Results show that the main influenced areas of light pollution are concentrated in the downtown and core areas of newly expanded areas with obvious deviation corrected like traditional serious light polluted regions (e.g., ports). In comparison, commercial–residential mixed areas and village-in-city show a high level of PELP. The proposed method better presents the extent of population exposure to light pollution at a fine-grid scale and the regional difference between different types of residential areas in a city.

## 1. Introduction

With the rapid development of the economy and the change to a modern city lifestyle, people’s demand for night lighting has increased in order to expand daytime activities. Due to the increasing and disorderly use of light at night, light pollution has increasingly become a prominent environmental issue. Back to the 1970s, scientists paid attention to light pollution at night against the background of global urbanization [1]. Meanwhile, the development of remote sensing has provided an effective way for observing urban light from space. Modern remote sensing technology has accelerated light pollution research [2]. An existing study reviewed the commonly used data including nighttime light (NTL) remote sensing images from the Defense Meteorological Satellite Program’s Operational Linescan System (DMSP/OLS), Visible Infrared Imaging Radiometer Suite (VIIRS) sensor on the Suomi National Polar-orbiting Partnership (NPP) satellites, and night light sensor from the Luojia 1-01 satellite [3].

Focused on exposure to light pollution, a large number of studies have confirmed the effects of light pollution on flora and fauna as well as human health. For instance, artificial light at night directly affects the growth and phenology of plants, and is likely to impact the behavior of herbivores [4]. Nighttime light, even very low-level illumination, can influence the habits of nocturnal animals (e.g., reducing the foraging activities of fruit-eating bats [5] and disrupting the life-histories in moths [6]). This affects the processes of reproduction in wild animals [7,8], which then also influences the animal population [9] and natural ecosystems [10]. Furthermore, nighttime light also has a great impact on sleep in free-living animals like cavity-nesting birds [11]. Research on nocturnal primates has shown that light pollution affects daily rhythms and behavior patterns such as the timing of seasonal estrus [12,13]. Several studies have indicated that the disturbance of circadian rhythms caused by light pollution is the main risk, especially given their influence on sleep at night in diurnal species as well as humans [14,15]. By linking human diseases and the order of light pollution, previous studies have found that there may be a causal relationship between light intensity and the incidence of breast cancer, according to disease samples and NTL remote sensing data [16,17]. In addition, there is evidence showing that the incidence of prostate cancer is significantly correlated with the intensity of light pollution [18].

Most studies on light pollution have directly used the digital number (DN) value or radiance from NTL remote sensing to express the degree of light pollution. Miguel et al. [19] combined NTL radiance from DMSP/OLS images, population, and public lighting consumption statistics to analyze the change of electricity consumption in Spain from 1992 to 2010, and found that the main light pollution comes from public street lighting. Applying a time series of DMSP/OLS satellite images, Bennie et al. [20] analyzed the contrasting trend in light pollution across Europe according to the change of brightness. In China, the world’s largest developing country, NTL remote sensing based light pollution monitoring and its spatial distribution trend from 1992 to 2012 was conducted according to the change of NTL radiance at night. One conclusion is that the Pearl River Delta in the southern coast of China is a region with relatively serious light pollution [21]. For smaller areas such as city-level scale analysis, some other data sources including high resolution commercial satellite images [22] and aerial photos [23] have been employed. The essence of these methods is the same as the use of NTL radiance to present light pollution in cities.

From previous studies, artificial night sky brightness perceived by various remote sensing sensors has been widely used to represent light pollution with less consideration of the impacts on densely populated or urban residential areas. However, the same illumination intensity may have different impacts when considering the location and population factor. In other words, there would be a certain deviation if evaluating light pollution only by using NTL radiance from the perspective of the impact of light pollution on human health.

In order to better illustrate the impact of light pollution on residential areas in cities, an index for measuring the level of population exposure to light pollution was proposed in this study. As ideal detectors of human behavior and population distribution [3,24], high-precision mobile data as well as high-resolution NTL data from Luojia 1-01 NTL remote sensing satellite were integrated for analyzing light pollution and its influence on populated areas at a fine grained resolution.

## 2. Study Area and Data

### 2.1. Study Area

Located in an area with serious light pollution, Shenzhen, a highly urbanized city in the Pearl River Delta region, China, was chosen as the study area (113.43 °E~114.38 °E; 22.24 °N~22.52 °N). As the forefront of China’s comprehensive reform, Shenzhen has become a major city and one of the economic centers in South China. Furthermore, it is also characterized as a center for science and technology innovation. The total land area of Shenzhen is 1,997.47 square kilometers. The permanent population has reached to 13,026,600 [25]. With rapid economic development, the electricity consumption in Shenzhen has increased by about six times in the past two decades [25].

### 2.2. Data

#### 2.2.1. High-Resolution NTL Remote Sensing Data

Luojia 1-01, the new generation NTL remote sensing data, were utilized in this study. The satellite was launched on June 2, 2018. The design of Luojia 1-01 stresses the perception of nighttime light at a finer spatial resolution of 130 m compared with traditional NTL remote sensing sensors like DMSP/OLS. It is a low orbit satellite with a repeat period of 15 days. The local visiting time is around 10:00 pm. According to previous studies, Luojia 1-01 data show great potential in monitoring artificial light pollution [3,26].

Considering the study area is located in South China where it is always cloudy and rainy, a cloud-free image acquired in September, 2018 was selected and adopted for the analysis.

#### 2.2.2. Mobile Big Data

The volume of mobile devices during night time in September, 2018 was collected to match the NTL remote sensing data. The location information of active mobile devices was collected through monitoring the mobile phone’s applications (apps). The active mobile device here means that the status of one or more monitored applications are activated at least once during a certain period. Given the activation of apps may not occur every day for some mobile devices, to reduce the omission of non-active devices, an accumulating number was adopted based on a month of cumulative observations of active mobile devices during night time from 10:00 pm to 6:00 am. Furthermore, the number of devices counted repeatedly (i.e., the same devices with apps activated more than once) was excluded. Higher positioning accuracy is provided by the mobile phone’s built-in global positioning system (GPS) unit. Considering the privacy policy, all location data were aggregated into grids. For gridded unit areas, their location information was encoded by the 7-bit GeoHash format, equivalent to the ground resolution of approximate 150 m. The mobile data were provided by the TalkingData company (Beijing, China), China’s leading third-party data provider.

**Figure 1:**
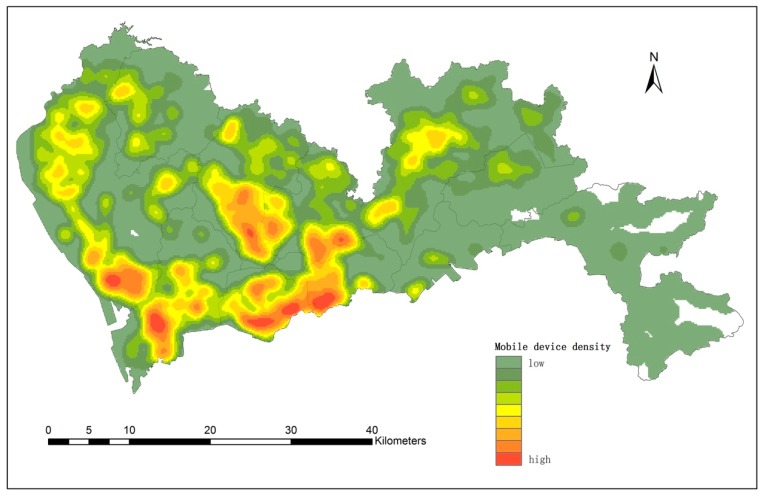
The distribution of active mobile devices during night time in Shenzhen (September, 2018).

After data cleansing, the original data encoded by GeoHash string were converted to a raster data format with geographical latitude–longitude coordinates, and clipped with the administrative boundary of Shenzhen municipality in ArcGIS software. Figure 1 shows the distribution of active mobile devices at night in Shenzhen.

#### 2.2.3. Auxiliary

**Figure 2:**
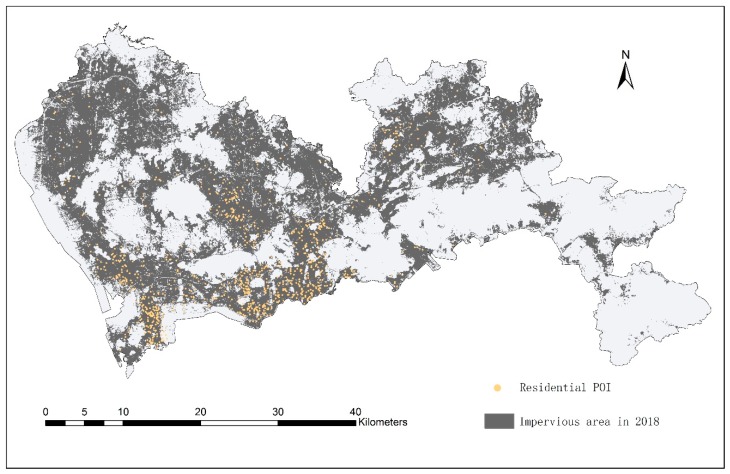
The distribution of the residential point of interest (POI) data in Shenzhen.

The auxiliary data include the fundamental geographical data, land cover data, and population statistics from the statistical yearbook. The land cover data of the urban impervious area in 2018 were adopted, which were obtained from Tsinghua University. Furthermore, in order to analyze the differences among various types of residential areas, the point of interest (POI) data of residential areas were acquired from the Gaode Map, one of the most popular map providers in the Chinese market. Nine hundred POIs covering different types of residential areas in Shenzhen were selected through the Gaode application programming interface (API). The attributes of POI data include the name of the place, category, address, geographic location information, code, etc. After preliminary processing such as the elimination of type misclassification and redundant points within an analysis unit (i.e., the size of a pixel), 871 samples were adopted. The spatial distribution of the residential samples is shown in Figure 2.

## 3. Methods

### 3.1. Data Pre-Processing

A geometric correction of the NTL remote sensing image was carried out by point-to-point registration according to the existing road vector data. For a better illustration, DN values on the remote sensing image were converted to radiance. According to [26], the following formula was adopted.

(1) $$r=5.2\times 10^{-6}\times DN^{3/2}$$

where r presents the radiance, the unit of which is nW·cm^−2^sr^−1^ and DN denotes the DN value for a pixel.

Considering the discrepancy between the spatial resolutions of multi-source data, NTL remote sensing and mobile data were unified to the same spatial resolution of 150 m by the nearest neighbor resampling method. Other auxiliary data such as urban impervious area and POI data were also processed to the same unit of analysis.

### 3.2. Gridding Population Distribution

Mobile big data can reflect the daily living activities of the people in the city. Mobile data at night revealed the population distribution as well as the population density in the grid. Zhao et al. [27] used economic density (i.e., Gross Domestic Product (GDP) per land area) to disaggregate the GDP from provincial to pixel level, with the disaggregation process being generalized by a linear model. Similarly, a linear model was adopted in this study to disaggregate Shenzhen’s total population based on the population distribution. Given the huge diversity in population density among administrative districts in Shenzhen, the transformation coefficient also varies for different districts. This coefficient is based on the correlation between the total population and the sum of active mobile devices in each district. From the annual statistics yearbook (2018), Table 1 shows the population by district and the corresponding number of mobile devices. Gridded population density ($P_{i}$) was simulated after data transformation by the following equation.

(2) $$P_{i}=\frac{P_{region}}{M_{region}}\times M_{i}$$

where the $P_{region}$ is the total population in the district where grid cell *i* is located; $M_{region}$ is the sum of active mobile devices in the same district; and $M_{i}$ is the number of active mobile devices in grid cell *i*.

### 3.3. Population Exposure to Light Pollution

To consider the impact of light pollution on humans, an index called the population exposure to light pollution (PELP) was proposed in this study. The index considers both NTL radiance and gridded population density and is defined by the formula as follows:

(3) $$PELP=R_{i}\times P_{i}$$

where $R_{i}$ is the NTL radiance at grid cell i; and $P_{i}$ is the population density represented by population in grid cell i.

### 3.4. Analyzing Spatial Pattern of Population Exposure To Light Pollution (PELP)

Gridded PELP values for the entire study area were calculated according to Equation (3). Regional differences in the 10 administrative districts are depicted. Both average and maximum values are compared among the districts. The analysis of variance (ANOVA) test was adopted to determine if there was a statistically significant difference between the averages.

Given that light pollution mainly affects the night living environment of settlements, the differences of the level of PELP in different residential areas were investigated. According to the properties of POI data, four categories were analyzed, namely high-rise dwelling (H), middle and low-rise dwelling (M), village-in-city (V), and dormitory (D). Furthermore, commercial–residential mixed (C) type was utilized as a contrast. Due to the existence of the misclassification of residential buildings in the POI data compared with field investigation, the 871 POI samples were reclassified into the above five classes. Table 2 illustrates the original type codes in Gaode Map and the corresponding types adopted in this study. Analysis of variance (ANOVA) was also adopted to test if there was a significant difference among the averages of those classes.

Furthermore, several areas traditionally considered as serious light polluted areas such as the airport and container terminals were analyzed as typical cases.

## 4. Results

### 4.1. Spatial Distribution of PELP in Shenzhen

**Figure 3:**
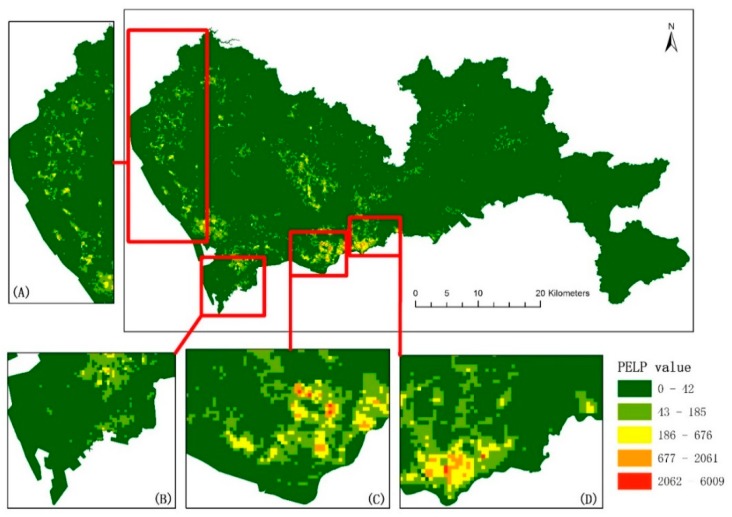
Spatial distribution of population exposure to light pollution (PELP) in Shenzhen. (**A**) Bao’an District, (**B**) Nanshan District, (**C**) Futian District, (**D**) Luohu District.

To conduct a street-scale analysis of light pollution, the PELP value for each grid cell was calculated. Considering that even very low NTL radiance would lead a higher PELP, if there is a huge population, a safety threshold for non-dangerous levels of brightness was adopted. The maximum NTL radiance of rural areas with vegetation based on the urban impervious area data were utilized as the threshold. Pixels with radiance below the threshold were not considered to be affected by light pollution. Figure 3 illustrates the spatial distribution of PELP in Shenzhen City. The PELP values were divided by 1000 in the following figures for a better display effect.

**Figure 4:**
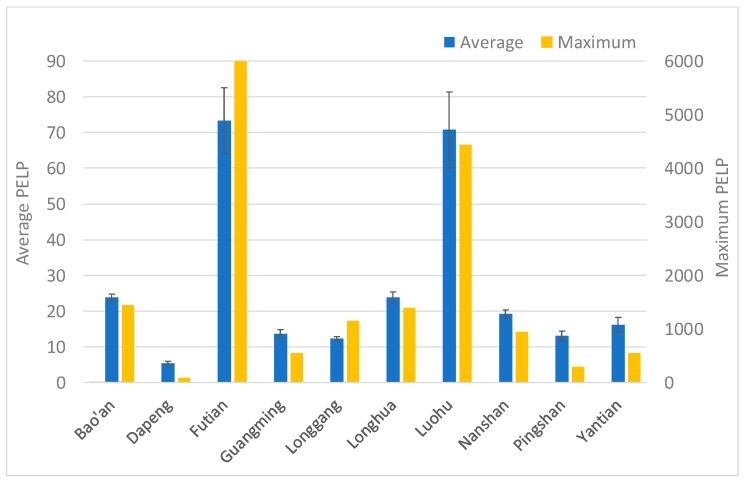
Regional difference of PELP by district.

For a comparison of different regions, Figure 4 shows the regional difference in PELP among the 10 administrative districts in Shenzhen. The 95% confidential intervals for the averages are shown as error bars in this figure. The ANOVA test results indicate a significant difference among districts (F-value = 187.12, *p* < 0.01). From the statistical results, Futian and Luohu Districts have high levels of population exposure to light pollution in terms of average PELP. Furthermore, newly developed regions including Bao’an and Longhua Districts also had a higher risk of exposure to light pollution.

### 4.2. The Impact of Light Pollution on Different Residential Areas

**Figure 5:**
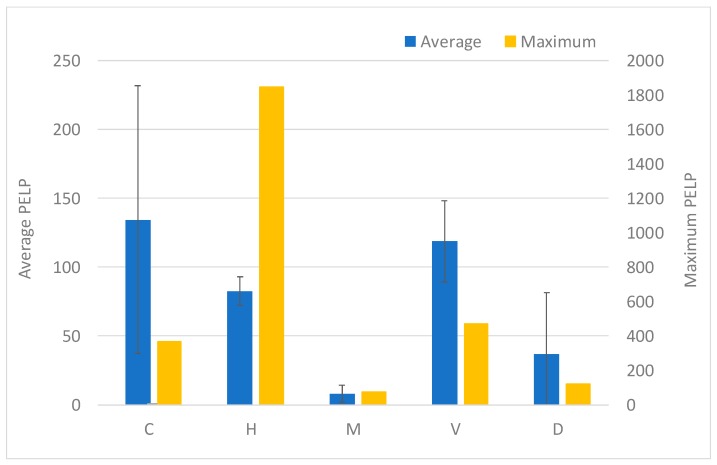
Difference of PELP by residential type.

According to the categorized residential areas, the maximum and average values of PELP for each type were calculated and are shown in Figure 5. The 95% confidential intervals for the averages are illustrated in Figure 5. The ANOVA test results indicate a significant difference among the five classes (F-value = 3.44, *p* < 0.01). Considering that the sample sizes of commercial–residential mixed and dormitory types were too small to present a statistical difference, an ANOVA test of the other three types was conducted, and a significant difference of average PELP among them was observed (F-value = 5.85, *p* < 0.01). From the results, village-in-city had the highest level of PELP in terms of average PELP. The average PELP of high-rise dwellings was larger than that of middle and low-rise dwelling. Furthermore, compared with the single function region of residential areas, commercial–residential mixed type also had a high level of PELP.

### 4.3. Typical Region Analysis

**Figure 6:**
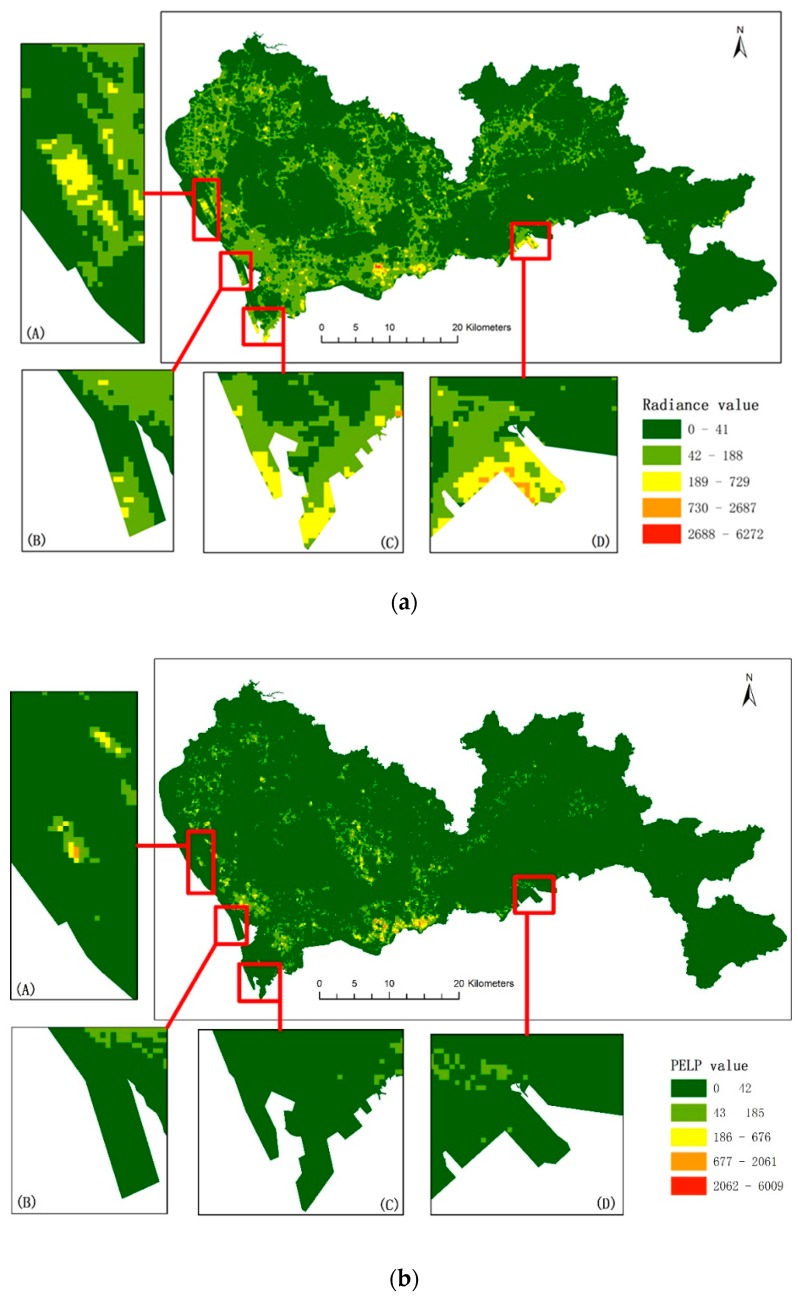
Comparison between the light pollution parameters based on nighttime light (NTL) radiance (**a**), and PELP (**b**), typical regions including (A) Shenzhen International Airport, (B) Dachanwan Port, (C) Shekou Cruise Port, and (D) Yantian Port.

Several typical regions traditionally considered to be seriously light polluted (Figure 6a) were selected including port areas (e.g., cruise port and container ports) and the airport. A qualitative comparative analysis was conducted by simply using NTL radiance and PELP. The results are shown in Figure 6 with the areas bounded in red lines. As for the areas of serious light pollution determined by traditional methods, there was no significant difference of the level of PELP compared with the surrounding areas except for the airport region (Figure 6b). Due to the presence of night flights, a certain number of passengers and ground crew appeared in this area. The level of PELP was higher than that of the surrounding areas and formed an island effect.

## 5. Discussion

### 5.1. NTL Radiance vs. PELP

Unlike the direct use of remote sensing radiance to express light pollution in cities, PELP obviously highlights the influence of nighttime light pollution on densely populated areas. When the population is small, even if the radiance is high, the index will be lower, whereas when the radiance and the population are both high, the index will be very high. For the case of Shenzhen, high exposure level areas were concentrated in Futian, Luohu, Nanshan, and Bao’an Districts, which had better economic development and high population density, while low exposure levels were located in the areas with low-level commercial development and low population density such as Yantian District and Dapeng New District. Both maximum and average values of PELP indicate that the regional difference was significant. Thanks to the introduction of the population factor, the proposed PELP index was more in line with the traditional definition of light pollution, that is, the influence of light pollution on people [1]. This is also more in line with people’s conventional understanding of the harm of nighttime light pollution. For example, high-level light illumination far away from residential areas has less impact on people, while the light from commercial centers or outdoor billboards around residential areas will cause more serious discomfort. From Figure 6, the port and airport areas had high level illumination. If using NTL radiance to assess light pollution, those areas have a high hazard level of light pollution, while in fact, the light in those areas have little influence since fewer residential areas are nearby. The deviation was significantly reduced when adopting PELP. Meanwhile, residential areas near the commercial areas like the commercial–residential mixed areas had a higher level of PELP (Figure 5).

It should be pointed out that a previous study presented a lit population indicator [27], whose calculation formula is very similar to that of the PELP proposed in this study. For a lit population, NTL radiance is treated as a measurement of GDP per capita, and multiple gridded population is used to calculate GDP density (i.e., GDP per pixel). Despite the same form, the physical meaning and calculation parameters of these two indices are different.

### 5.2. Differences in Residential Areas

The application of high-resolution NTL remote sensing data and high-precision positioning mobile data makes a street-scale analysis of the level of PELP possible. Accordingly, investigation of the differences among different residential areas could be carried out. From the perspective of sample distribution, most POI samples belonged to high-rise dwelling and presents as the most common type of residential buildings in Shenzhen. Its level of PELP was higher than that of middle and low-rise dwellings, but lower than that of village-in-city. Middle and low-rise dwellings were concentrated in the suburbs or those areas far away from downtown, where the environment is quiet and the population density is relatively low. The village-in-city is another case with high dense population and very high brightness during the night. As a special case in the rapid development of urbanization, the village-in-city consists of many rental rooms and accommodates a large number of the floating population like migrant workers. As such, the level of PELP can be rather high.

In contrast, the commercial–residential mixed type had a higher level of PELP, because the Internet, information technology (IT), and related industries in Shenzhen are highly developed and night life is relatively rich in those areas. The commercial–residential mixed type implies a high density population, where there are high intensity lighting facilities. Therefore, according to the PELP index, the differences between residential types are exaggerated. It should be noted that the smaller sample size of commercial–residential mixed and dormitory types may cause a deviation to some extent.

### 5.3. Limitation and Known Issues

For a wide scope of light pollution study, three forms of light pollution are referred to, namely sky glow (or upward light flux), glare, and light trespass [28]. Current studies on remote sensing light pollution are focused on upward light flux due to the limitation of observations. Therefore, the light pollution at night discussed in this study mainly refers to this issue.

In addition, the estimation model of population density in a grid was based on mobile data and was adjusted by the population in statistics. For the statistical yearbook, the only permanent population is considered for the official census count, and does not include the floating population. Considering Shenzhen is a migrant city, possible uncertainties may come from the deviation in population density estimation. Furthermore, the modifiable areal unit problem (MAUP), which is well-known for scale and zonation issues [29,30] may occur when aggregating point data (e.g., mobile data and population) into areal unit (e.g., grid or pixel). This means that it may yield different analysis results when aggregating and analyzing the population distribution at different spatial resolutions.

## 6. Conclusions

This study proposed an index to quantify the level of population exposure to light pollution based on integrating high-resolution NTL remote sensing and high-precision mobile phone data. The spatial distribution pattern of light pollution in Shenzhen was illustrated at a street scale level. The regional differences between administrative districts as well as different types of residential areas were analyzed. By introducing the population factor, it turns out that the proposed method could well reflect the impact of light pollution on populated areas, and significantly reduce the deviation such as light polluted regions with low-density population. A large proportion of young people and city vitality has made the nighttime light pollution become worse. Among the different types of residential areas, the commercial–residential mixed type had a high level of population exposure to light pollution. Furthermore, the village-in-city’s level of PELP was also higher. This study had a positive promoting effect on urban construction and energy consumption in smart city management. For a further study, analysis of multi-temporal NTL remote sensing and mobile data could be conducted to monitor the dynamics of population exposure to light pollution.

## Figures and Tables

**Table 1:** Actual population statistics and the volume of active mobile devices in different districts.

District *	Actual Population	Number of Mobile Devices
Bao’an	3,149,000	1,012,949
Dapeng	146,100	29,647
Futian	1,561,200	544,395
Guangming	596,800	185,975
Longgang	2,278,900	955,800
Longhua	1,603,700	621,306
Luohu	1,027,200	370,078
Nanshan	1,424,600	583,802
Pingshan	428,000	66,134
Yantian	237,200	45,472

Note: Population statistics is updated to the end of the previous year. * Statistics of Shen-Shan Special Cooperation Zone (enclaves) is not included.

**Table 2:** A look-up table for residential types adopted in this study and original type codes in Gaode Map point of interest (POI) data.

Origin Code in Gaode Map	Type of Residential Areas	Type Code	Sample Size
120201|120203|120302	Commercial-residential mixed (C)	0	8
120203|120300|120301|120302|120303	High-rise dwelling (H)	1	761
120300|120301|120302	Middle and low-rise dwelling (M)	2	33
120300|120302	Village-in-city (V)	3	62
120303	Dormitory (D)	4	7
